# Chronic Kidney Disease of Unknown Origin: Think Beyond Common Etiologies

**DOI:** 10.7759/cureus.38939

**Published:** 2023-05-12

**Authors:** Alokita Trivedi, Sunil Kumar

**Affiliations:** 1 Department of Medicine, Jawaharlal Nehru Medical College, Wardha, IND

**Keywords:** hiv, hypertension, environment, diabetes, ckdu

## Abstract

Chronic kidney disease has an increased health impact on a global scale, with the most common etiologies being hypertension and diabetes. It is most frequently linked to noncommunicable conditions, including diabetes and hypertension, among high-income nations. However, it has a couple of new potential etiologies in low- and middle-income countries, many of which are yet unknown, including viral infections and environmental toxins. The phrase "CKD of unknown etiology" (CKDu) has been used to refer to CKD that is not caused by a typical risk factor such as diabetes, high blood pressure, or HIV.

Environmental variables have been investigated as potential contributors to CKDu, including heavy metal exposure, elevated seasonal temperatures, pesticide use, mycotoxins, contamination of water supplies, and snake bites. Furthermore, the underlying causes have not been definitively established in the majority of areas and identifying serious health consequences across different international contexts and populations may be crucial for comprehending and avoiding CKDu.

## Introduction and background

Chronic kidney disease is an increasing public health issue of worldwide scope. Although specific estimates are not readily available from these nations, the prevalence is rising not only in industrialized nations but also in low- and middle-income nations. DM and hypertension are two primary causes of chronic kidney disease (CKD) in many countries, although other factors that are less well defined, referred to as "nontraditional" by some nations, can also contribute to its onset [1].

While efforts are being made by the international community to combat the CKD epidemic in urban areas, CKD of undetermined etiology (CKDu) is gradually being documented in remote, primarily rural locales in many parts of the world. Clinically, CKDu is diagnosed after excluding all the known causes of CKD. This disease has been reported in young and middle-aged adults, mainly in agricultural and manual laborers (males) doing physical work for their livelihood. The patients have minimal or no proteinuria.

Usually, CKDu is considered or suspected on the basis of eGFR less than 60 mL/min/1.73 m2 by the CKD-EPI formula and/or urine protein 1 plus or more by dipstick. Patients should not have a history of diabetes mellitus or taking oral hypoglycemic agents, polycystic kidney disease, renal stones, or obstructive pathology like benign prostrate hypertrophy, vasculitis, lupus, or congenital kidney disease. These patients should not have blood pressures greater than 140/90 in stages 1, 2, and more than 160/100 in stages 3, 4, and 5 of CKD, as well as not be on two or more types of antihypertensive treatment [1].

The disease begins insidiously, and there are few or no symptoms till the disease is fairly advanced. Some of the markers that can identify CKD in its early stages are not easily available. With CKDu, most of the early manifestations are associated with tubular abnormalities and alterations in urinary sediment before there is clinically evident proteinuria/albuminuria or a fall in glomerular filtration rate (GFR). [2,3] There are further difficulties in estimating GFR reliably, and even then, by the time there is a definite fall in estimated glomerular filtration rate (eGFR), the disease would be clearly advanced and irreversible. These are some of the problems with diagnosing CKDu. Characteristics of CKDu may vary in different geographic locations, like in Central America (rural and coastal regions of Nicaragua and El Salvador), 20-40 years of age, predominantly male, are affected. They were involved in occupations like sugarcane, bananas, subsistence farming, and mining. In Sri Lanka, people aged 30 to 50 were more affected in rural areas and the North Central Province. Surprisingly, females suffered more than males. Paddy field workers and Chena farmers were the main occupations [1,3]. In India, the rural Uddanam area in Andhra Pradesh, Narasinghapur block in Odisha, Akola district in Maharashtra, and Canacona district in Goa were more affected. In the third and fourth decades, individuals involved in coconut, cashew, and rice farming were more affected. Characteristic clinical features were a progressive fall in eGFR, minimal proteinuria (usually absent), generally <1 g/day, hematuria, bland urine sediment, normal blood pressure, and small, shrunken, symmetrical kidneys. Low altitude, strenuous work, sugarcane cutting, heat stress, NSAIDs, high consumption of sugary drinks, pesticides, leptospirosis, heavy metals like cadmium and arsenic, pesticides, illicit liquor, hantavirus infection, ground water consumption, silica, strontium exposure, lead, and heat stress were the postulated hypotheses in all these regions [1,3].

Method

**Figure 1:**
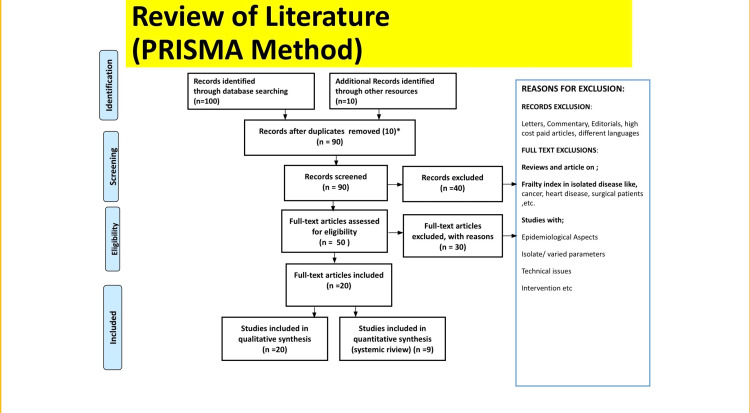
Research methodology by PRISMA method

A literature search in English was conducted using the electronic databases PubMed, MEDLINE, Embase, Google Scholar, and ResearchGate. A separate search was done on it as well. The search terms were “CKD” OR “Chronic renal disease”, chronic kidney disease, OR “unknown origin,” OR “occupation,” OR “rural areas,” OR “diet,” OR “lifestyle.” The archiving of relevant papers was supported by the writers’ personal knowledge and experience in the field. Articles that match the following criteria are included in this review: studies in English are included; studies from the previous 10 years are included as well; and studies devoted entirely to CKD, nutrition, and lifestyle modification are included. Research methodology using the PRISMA method has been highlighted in Figure 1.

## Review

Common clinical traits distinguish CKDu from several of the recognized causes of CKD and serve to characterize it. The condition is typically found in youth and middle-aged males who undertake physically demanding jobs for a living, like daily labor and farming, throughout all reported places in the world. There is little to no proteinuria among the patients. Patients with CKDu are often non-diabetic and also have normal blood pressure or very modestly elevated blood pressure [1].

A mysterious epidemic of chronic kidney disease of unknown origin

One of the most difficult health risks in today's reality is one with unidentified root causes. Chronic kidney disease of unexplained etiology, an enigmatic epidemic of renal disease, is seen in various parts of the world. The majority of people who are affected by CKDu are young and middle-aged, with little male dominance, and it is mostly unrelated to known risk factors like DM and hypertension. People who live in rural areas, particularly those who work in agriculture, are more likely to experience it. Contrary to other serious illnesses like diabetes and AIDS, the proportion of people with CKD, as well as those who require dialysis and kidney replacement, is rising every year [2].

Is CKDu related to environmental urbanization?

The elderly, obesity, and diabetes may be contributing factors to an increase in chronic kidney disease (CKD) globally, but this does not explain the environmental cluster of CKDu. These environmental clusters of CKD have been reported in countries such as Sri Lanka, Central and Latin American countries, and regions within Egypt and India. One study from Belfast, UK, investigated environmental factors as a cause of CKDu. This study revealed increased elemental concentrations of potentially toxic elements in soils due to urbanization. The constituent proportions of lead (Pb), cadmium (Cd), mercury (Hg), molybdenum (Mo), tin (Sn), antimony (Sb), and arsenic (As) had been reported to have the strongest connection with CKDu [3].

Authentic diseases that couldn't be addressed or related to usual causes have emerged, and these diseases have been related to climatic and global warming considerations [4].

Several causes of renal dysfunction can be due to the eating practices and toxins found in the meals used by people living in polluted areas. Chromium and lead were present in rice, freshwater fish, fruits and vegetables, and legume vegetables. [5]. However, more research is needed to explain the relationship of CKDu with levels of elemental toxins and the historical legacy of anthropogenic contamination.

Chronic kidney disease of unknown origin: glyphosate's symbiotic toxic effects in conjunction with various components

The condition has doubled in Sri Lanka over the past four or five years since it was discovered in the North Central area in the middle of the 1990s. People in pastoral areas who lack the well-known CKD possible risks are the group most typically affected by the disease. Sri Lanka is not the only country where CKDu is prevalent; health professionals have also noted cases in Mexico, Nicaragua, El Salvador, and the Indian state of Andhra Pradesh [6].

Dehydration brought on by heat exhaustion, deadly metals like cadmium and arsenic, fluoride, low selenium, damaging cyanobacteria, nutrient-poor food, and mycotoxins from mold exposure are a few of these causes. Additionally, contact with pesticides and fertilizers, specifically glyphosate and paraquat, is probably a contributing component and might even be the main one. The additive health implications of glyphosate, along with sensitivity to several poisons, particularly paraquat, and strenuous physical activity in lowland tropical locations with constant warm altitudes, may cause kidney impairment similar to CKDu in Sri Lanka [6].

Considering serum retinol-binding protein 4 (RBP4) as an added biomarker for the assessment of CKDu

For a thorough assessment of illnesses, and consequently, in CKD, serum proteins, specifically those with a profile of markers representing various features of the disease, are helpful. It was determined that the RBP4: S.cr ratio could distinguish CKDu from CKD with >70% sensitivity and specificity. As a result, it might be employed in the assessment of CKD's tubular interstitial participation [7].

The status of being immunocompromised, given that the immune system cannot effectively resist the lethal extreme acute pulmonary crisis coronavirus 2, is frequently linked to severe coronavirus infection (SARS-CoV-2). Patients who have several comorbid conditions, notably DM, hypertension, and CKD, along with those taking immunosuppressive drugs or receiving chemotherapy, are much more likely to contract the disease during the current epidemic and experience negative results [8].

Spread and factors affecting CKDu

Worldwide, chronic kidney disease (CKD) is responsible for major morbidity and mortality. The major frequent sources of CKD are DM and hypertension. CKDu, a subtype of CKD lacking any well-established general factors, is another condition that is being more widely documented in various parts of the world, including Central America, Sri Lanka, and India. They are mainly seen in rural fields where crops like sugarcane and coconut are grown extensively. Men and young people are more likely to acquire CKDu [9].

It primarily harms people with a lower socioeconomic background. Exposure to silica, arsenic, and fluoride is linked to a higher occurrence of CKDu. It is unknown but not impossible that heat stress plays a role in dehydration-related CKD. In some situations, it has also been discovered that CKDu is related to mycotoxins like aflatoxins and ochratoxins. According to research, CKDu has a substantially favorable relationship with agricultural pesticides such as HCH, Endosulfan, Alachlor, and Pendimethalin. Hantavirus and Leptospirosis infections may potentially play a part in the extreme febrile stages of CKDu. Research findings on CKDu have not, nonetheless, provided solid proof of its etiology or risk factors [9].

The use of new renal biomarkers for diagnosing chronic kidney disease with unknown causes

The non-communicable disorder with high morbidity and mortality associated with chronic kidney disease (CKD) is widespread throughout the world. Although it is often linked to diabetes and hypertension, CKD of undetermined etiology (CKDu) has emerged in various tropical agricultural societies over the past 20 years and has destroyed thousands of lives. When CKDu is present, renal function gradually declines over time without any warning signs, potentially leading to full renal failure and death. Youthful adult males from lower socio-economic groups are especially affected. The creation of stronger diagnostic and therapeutic techniques is crucial since early recognition can efficiently slow the advancement of the ailment. In contrast to existing boundaries, novel biomarkers with better sensitivity and specificity are now being proposed as prospective strategies for initial disease evaluation in contrast to existing biomarkers. In this study, new novel biomarkers for CKD assessment are summarized, along with their ongoing efficacy and diagnostic potential for CKDu screening in hospital studies of various CKDu-affected regions [10].

The burden of CKD has escalated substantially, and chronic kidney disease of unknown etiology greatly worsens CKD's effects at the national level (CKDu). Issues frequently start to surface in the final stages of the sickness, which is described as an incurable, slowly deteriorating disorder. The causative agents are said to be complicated and comprise climatic, familial, occupational, and societal factors. Prolonged tubulointerstitial nephritis is the main histological characteristic [11].

The gut microbiome gets altered by prebiotics and probiotics, which slow the development of CKDu. Regarding a small group of individuals, soda bicarbonate treatment is inexpensive and effective in halting the advancement of CKDu. Contrary to various sources of CKD, culinary adjustments to combat dehydration are required [12].

Mysterious causes of chronic kidney disease in agricultural communities

The incidence of chronic kidney disease with an unclear cause in farmland populations has recently been observed in Central America, Egypt, India, and Sri Lanka, mostly among male farm workers. It was often higher among workers between the ages of 20 and 50, and it varied according to the local economy and altitude. In between 57.4 and 66.7 percent of cases, the cause was not known. Chronic tubulointerstitial nephritis was the primary histological diagnosis. Associations with a family history of chronic renal disease, exposure to agrochemicals, dehydration, hypertension, and homebrewing alcohol consumption have all been recorded [13].

A "magnet" of chronic renal disease with an unknown cause has been identified as the Bade village in Northern Yobe State. Despite numerous attempts to find the condition's genuine cause in the region, the exact etiology remained a mystery. Previous research suggests that exposure to heavy metals through various channels may act as a disease "magnet" [14].

According to Sri Lankan standards, various criteria, like the levels of Mg, Na, K, sulphate, and phosphate, are below the acceptable range. Arsenic is present in 23.7% of the specimens (mean value: 0.0054), and cadmium is not present in any of the samples. Using linear regression, it was found that CKDu and water quality factors were related [15].

In the remote region of Bangladesh, the occurrence of chronic renal disease is 10.2%. About a quarter (23%) of these participants likely fell into the CKDu classification [16].

According to a cross-sectional analysis under the DEGREE protocol conducted in Peru, along with the standard danger aspects for CKD, environmental situations for CKDu (such as working in farms, water sources, hot flashes, and pesticide susceptibility) were assessed. In both villages and urban sectors of Northern Peru, a minimal community prevalence of reduced eGFR (as a stand-in for CKDu) was discovered [17].

Genetic screening to diagnose kidney disease with no known cause

Even after a patient has undergone a thorough renal evaluation, the cause of chronic kidney disease is frequently unknown. Genetic analysis promises to speed up diagnosis and enable customized treatment in cases of kidney disease with an unknown etiology. According to a recent study employing genetic testing, Mendelian etiologies account for roughly 20% of cases of kidney illness with unclear etiologies. While genetic testing has several benefits, including the capacity to lead targeted workups, uncover extrarenal disease, counsel patients and families, and refocus care, significant disadvantages and risks must be considered [18].

An epidemiologic review on the relationship between exposure to pesticides and undiagnosed chronic kidney illness

CKDu is more common in the agrarian industries among men, even if it can also be prevalent in women. Consequently, exposure to pesticides was previously thought to be the cause, but today, prolonged heat stress and dehydration are recognized as significant etiologic factors. There is little evidence from recent studies to indicate a connection between pesticide use and localized CKDu epidemics, but a role for nephrotoxic agrochemicals cannot be fully ruled out given how inadequately most studies quantify pesticide exposure [19].

WHO established permissible levels for lead (Pb), cadmium (Cd), and arsenic (As) concentrations in the well water. All rice specimens, except Pb, contained levels of many other heavy metals (loid) below the WHO's maximum permissible limits (0.02 mg/kg). A study of the hazardous index of Cd, As, and Pb revealed that 26% of the rice specimens could place this citizenry's well-being in jeopardy via rice intake. Twenty-three percent (23%) of the rice specimens analyzed violated the acceptable range for TDI of Pb [20].

According to a study in the Uddanum region of Srikakulam, Andhra Pradesh, India, the amount of food consumed each day exceeded safety limits for lead, chromium, and manganese exceeded safety limits. It is recommendable to lessen vulnerability to heavy metals by implementing farmland practices and dietary public health initiatives [21].

Dissolved organic matter (DOM) in CKDu groundwater differed from DOM in non-CKDu groundwater in that it appeared more unsaturated, oxidized, physiologically refractory, and elevated in weight, and it possessed more aromatics (AS, 15.4%) but fewer aliphatics (AL, 8.2%), which was likely due to the refilling of fresh water [22].

Chronic kidney disease of unknown origin in Sri Lanka

According to the research, hot stress may not seem to be the exact cause of CKDu in Sri Lanka, indicating that vocational dangers such as exposure to agrochemicals are more certain to be linked to renal disease [23].

CKDu is an international health issue that mostly affects rural farmers in Mesoamerica and South Asia who are of low socio-economic status. The majority of sufferers are just confirmed with CKDu at the terminal stage of their renal illness due to the scarcity of usual symptoms like diabetes and hypertension and the absence of molecular markers. The urgent demand for biomarkers for earliest identification has been brought to light by the world's resilience to temperature increases and chemical pollutants [24].

Anemia is a common complication for patients with chronic kidney disease (CKD), which negatively impacts their prognosis [25]. Studies on anemia phenotypic traits in CKDu are, unfortunately, scarce. Anemia in CKDu is linked to disease incidence. The variations in anatomical subtypes of anemia within CKDu rounds might be described by a bigger pool with matching shares of subjects in the corresponding CKDu stages [25].

Evaluation of studies on chronic kidney disease of unknown etiology (CKDu) carried out in Asia and Latin America

The studies used to identify the warning variables linked to CKDu have not been standardized and have often had a narrow territorial focus. Regional differences in CKDu's scientific focus are evident. A more unified strategy for CKDu studies would lead to a better knowledge of the adverse outcomes connected to CKDu and how they differ in different areas [26].

Agrochemicals and related byproducts were found in large amounts in both glomerular filtrate and blood. AMP-activated protein kinase (AMPK), protein kinase-C, glutaminase, apoptotic signaling kinase-1, acetylcholinesterase, cytochrome P450, and glutathione-S transferase have been implicated in the course of CKDu, according to findings from the literature [27].

Because of the lack of slightly earlier warning indications and symptoms, CKDu cannot be diagnosed unless it has developed irreversibly. To give individuals a healthier prognosis, studies are done to determine the origins of the illness. The studies have made it possible to see more parallels and a few discrepancies among the CKDu occurrences documented in other nations, aiding researchers and healthcare workers in the early diagnosis and effective treatment of CKDu [28].

An unknown etiology chronic kidney disease clinicopathological and biochemical profile at a tertiary care rural hospital in Central India

A study was conducted in the Vidarbha region of Maharashtra in the years 2019-20 among the farmers. The major crops grown here are wheat, jowar, cotton, and pulses. Farmers frequently become dehydrated because of their repetitive exposure to intense heat and the hot temperature in Vidarbha, which causes them to sweat excessively and makes it difficult for them to drink enough water. In the study, habit history showed that 24% of the participants had an ongoing pattern of drinking alcohol, and 12% tended to smoke or chew tobacco. There was no substantial family history of CKDU in the research. In contrast to the Yavatmal trial, when only five of the 19 patients had hypertension and were using antihypertensive drugs, the study's newly found hypertensive cases were 10%, and instances of diabetes mellitus were 0%. On renal biopsy, tubulo-nephritis with the absence of immune deposits were observed. Twenty percent of patients showed clinical symptoms like nausea, loss of appetite, arm and leg swelling, vomiting, and muscle cramps. Neurological symptoms were observed in 20% of patients [29].

Individuals suffering from chronic kidney disease (CKD) may identify preclinical atherosclerosis using the ankle-brachial index (ABI), a nonsurgical technique. There aren't many studies on this in India, particularly in rural areas. In primary care settings, peripheral arterial disease (PAD) is important in CKD and is easily identified using the ABI approach via the palpatory method. When utilized as a preliminary assessment strategy for the early detection of individuals at significant risk of mortality and morbidity like CKD, the palpatory method provides a valid, straightforward, and objective strategy that may be performed in the doctor's office [30].

Due to their elevated likelihood of developing cardiovascular disease (CVD), individuals who have chronic kidney disease (CKD) experience higher rates of mortality, morbidity, and expenditures on healthcare. The primary cardiovascular disease (CVD) risk variables of atherosclerosis have been demonstrated to correlate with carotid intimal medial thickness (CIMT). CIMT and additional risk factors such as age, gender, and diabetes mellitus were independent variables, while HTN, consumption of alcohol, smoking, and dyslipidemia were all dependent variables. B-mode ultrasonography evaluation of the common carotid artery's CIMT was discovered to be an effective noninvasive tool for observing the arterial walls and keeping track of the initial phases of the process of atherosclerosis. The measurement of CIMT can also be used to evaluate the outcomes of medical treatments for atherosclerosis and is useful in diagnostic decision-making regarding the optimal course of treatment for those with carotid artery stenosis, whether surgical or medical [31].

Various studies regarding CKDu and its possible pathogenesis, with observations and conclusions, have been highlighted in Table 1.

**Table 1:** Various studies regarding CKDu with its observations and conclusions

Author and title of the study	Observation	Conclusion
Anupama YJ et al., 2019, "Chronic Kidney Disease of Unknown Etiology: Case Definition for India - A Perspective" [1].	The clinicopathologic aspects of CKDu were distinctive, and its multifaceted etiology was mostly attributed to numerous environmental contaminants.	A thorough definition was required to perfectly determine the cases.
Paidi G et al., 2021, "Chronic Kidney Disease of Unknown Origin: A Mysterious Epidemic" [2].	The majority of people who were affected by CKDu are youth and middle-aged, with a little male preponderance, and it was independent of identified risk factors like diabetes and hypertension. People who live in towns and villages, particularly those who worked in agriculture, were more likely to experience it. In comparison to other chronic illnesses like diabetes and AIDS, the proportion of people with chronic kidney disease, as well as those who require dialysis and renal replacement, is rising every year.	This extensive analysis was conducted to emphasize some inadequately recognized epidemiologic risk variables, including the progression of the disease because a definitive etiology for CKDu had not been suggested.
McKinley JM et al., 2020, "Chronic kidney disease of unknown origin is associated with environmental urbanization in Belfast, UK" [3].	With significant levels of 0.001, 0.01, and 0.001, correspondingly, it was shown that CKD, as well as the societal deprivation parameters of job, income, and education—which had been employed as proxies for socioeconomic determinants like smoking—had a mathematically significant link. The elemental ratios of Cr/Ni and As/Mo had been observed to have the strongest connection with CKDu adopting three different regression techniques (linear, generalized linear, and Tweedie models).	The study shed light on the rising prevalence of CKD worldwide, along with, in particular, manmade and ecological variables that might be associated with the disease, notably environmental PTEs associated with urbanization.
Geladari E et al., 2021, "Failing kidneys in a failing planet; CKD of unknown origin" [4].	Over 20,000 people died from chronic renal disease, a 177% increase in death rates. Intermittent heat and dehydration were first seen to have a significant effect on the development of chronic renal disease in animals. Younger male agricultural laborers in South Asia and Central America frequently expose themselves to high heat, which causes kidney diseases.	According to the research, MeN is a renal disorder that has not yet been recognized, causing impairment in both the tubulointerstitial and glomerular compartments.
Dayananda et al., 2022, "Determination of relationships between heavy metals in food and CKDu prevalence in Rideemaliyadda, Sri Lanka" [5].	The maximum levels of lead (7.52 0.19 mg/kg) and chromium (6.68 0.15 mg/kg) were found in green vegetables and rice, respectively. Each of the metal concentrations in question in the inland marine specimens was found to be below the specified PTWI values.	The people of Rideemaliyadda-south GND cultivate their very own rice, which is the most significant and proportionately the most eaten item of food in their daily diet. The key factor contributing to the incidence of CKDu in the region might be contacted with hazardous metals, particularly lead and chromium, via rice eating.
Gunatilake S et al., 2019, "Glyphosate's Synergistic Toxicity in Combination with Other Factors as a Cause of Chronic Kidney Disease of Unknown Origin" [6].	The etiology of CKDu may be caused by a variety of factors, not just one, according to a worldwide search for the source of the disease. Several of these factors included heat exhaustion that causes dehydration, poisonous metals like cadmium and arsenic, fluoride, little selenium, hazardous cyanobacteria, nutrient-wise insufficient diets, and mycotoxins from mold exposure. In addition, physical labor in the consistently hot lowland tropical regions may cause renal damage consistent with CKDu in Sri Lanka.	The cumulative health impacts of glyphosate, in addition to sensitivity to various other pollutants, particularly paraquat, and strenuous physical activity in lowland tropical locations with constant high temperatures, may have caused kidney impairment similar to CKDu in Sri Lanka.
Stalin P et al., 2020, "Distribution and Determinants of Chronic Kidney Disease of Unknown Etiology: A Brief Overview" [9].	Pesticides utilized in farming, such as HCH, Endosulfan, Alachlor, and Pendimethalin, show a strong positive correlation with CKDu. Hantavirus and Leptospirosis infections may potentially play a part in the acute febrile phase of CKDu.	Investigations into CKDu have not provided solid proof of its etiology or risk factors. Therefore, to investigate the etiology and pathophysiology of CKDu in varied contexts, larger research using improved methods must be carried out.
Gunasekara TDKSC et al., 2020, "The Utility of Novel Renal Biomarkers in Assessment of Chronic Kidney Disease of Unknown Etiology (CKDu): A Review" [10].	The creation of enhanced detection, as well as management techniques, is crucial since timely identification may successfully slow the advancement of the disease. Novel biomarkers with better sensitivity and specificity were proposed as prospective instruments for early disease diagnostics in comparison with traditional biomarkers.	Increased utilization of indicators could shed important light on the locations and procedures behind the renal injury, in addition to helping to further confirm their diagnostic utility and establish possible prognostic significance.
Abdissa D et al., 2020, "Purposeful Review to Identify Risk Factors, Epidemiology, Clinical Features, Treatment and Prevention of Chronic Kidney Disease of Unknown Etiology" [11].	Chronic tubulointerstitial nephritis was the main histological characteristic. It primarily affects people with low-income families who are working-age, largely healthy, and undertake demanding labor in hot, humid environments throughout diverse tropical regions worldwide.	Morbidity and mortality must be reduced through early detection and suitable therapies at the earliest opportunity.
Anupama et al., 2019, "Beneficial dietary options available that slow down progression of CKDu include prebiotics, probiotics, soda bicarbonate" [12].	Contrary to other causes of CKD, nutritional measures to avoid dehydration were required. These included supplying clean drinking water, a proper protein-containing diet with enough calories, and a calibrated consumption of salt to minimize hypotension.	Prebiotics and probiotics modify the gut microbiota, which lowers the release of uremic toxins and slows the course of CKD. Treatment with soda bicarbonate reduces the course of CKD. To prevent dehydration, malnutrition, and uremic symptoms, dietary monitoring, and tailored nutritional therapy, according to the urgent demands of the susceptible CKDu patients, were of the utmost importance.
Almaguer M et al., 2014, "Chronic kidney disease of unknown aetiology in agricultural communities" [13].	17.9% to 21.1% of people had proven chronic renal disease. According to one single serum creatinine measurement, the incidence of decreased glomerular filtration (60 mL/min/1.73 m2 body surface area) was 0%-67% in males and 0%-57% in women. The incidence was often higher among male farm laborers between the ages of 20 and 50, and it varied according to the local economy and altitude. In 57.4% to 66.7% of cases, the source was not known.	There wasn't much support for a single reason, and several ecological, occupational, and societal factors were at play.
Prabagar et al., 2020, "Impact of water quality on Chronic Kidney Disease of unknown etiology (CKDu) in Thunukkai Division in Mullaitivu District, Sri Lanka" [15].	The bulk of patients that were observed were smokers and drinkers, with 80% of the men being in the 50–70 age range. The most prevalent type of water supply in the area was a dug well.	The presence of CKDu in the Thunukkai region of Sri Lanka's Mullaitivu District may have been positively correlated with overall soluble solids plus arsenic in consuming water.
Hays T et al., 2021, "Genetic testing for kidney disease of unknown etiology" [18].	Mendelian etiologies account for about 20% of cases of kidney illness with unknown etiologies, according to recent research using genetic testing.	Despite the fact that genetic testing offered several advantages, such as the ability to adapt medicine, guide focused workups, identify extrarenal disease, counsel patients and their families, and refocus care, some substantial drawbacks and hazards that must be taken into account.
Valcke M et al., 2017, "Pesticide exposures and chronic kidney disease of unknown etiology" [19].	In agricultural jobs, correlations between pesticides as well as other concurrent exposures, particularly heat stress and dehydration, have not been studied.	Repeated contact with pesticides or agrochemicals, heat stress and heavy metal contamination were the main contributors to the rising incidence of CKDu worldwide. According to numerous writers, young men are more likely than women to get severe CKDu. Additionally, it is believed that the people of CKDu come from relatively poor socioeconomic classes, work in the mining or farming industries, and are frequently exposed to high temperatures.
Kulathunga et al., 2022, "Health Risk Assessment From Heavy Metals Derived From Drinking Water and Rice, and Correlation With CKDu" [20].	Heavy metal (loid) contributions to groundwater TDI were very small and unlikely to result in health risks, including CKDu.	Except for lead in rice, groundwater, and rice in Sri Lanka's CKDu disease-prone regions were safe and free of hazardous levels of heavy metals (loids).
Gupta et al., 2022, "Preliminary assessment of heavy metals intake via food in CKDu affected Uddanam region of Srikakulam, Andhra Pradesh, India" [21].	The National Institute of Nutrition and the Institute of Medicine's recommended intake levels were used to evaluate the daily intake of food. The intake of chromium, lead, and manganese exceeded permissible levels.	It was recommended to decrease exposure to heavy metals by implementing agricultural practices and dietary public health initiatives.
Kolli et al., 2022, "Molecular Monitoring for CKDu in Tropical Agricultural Communities: Unraveling the Mysteries of a Global Kidney Disease Epidemic" [24].	Specimens were pooled from every community and presented data that clearly suggested pooling as an affordable solution because the pooled samples accurately and sensitively represented the people within.	Proteomic data collected from the entire community will demonstrate the existence of early detection indicators required to develop prompt and efficient treatment plans.
Redmon et al., 2021, "A comparative review: Chronic Kidney Disease of unknown etiology (CKDu) research conducted in Latin America versus Asia" [26].	Research on putative CKDu risk factors in Latin America has mostly concentrated on dehydration/heat stress, and the use of agrochemical products. Particularly in Latin America, more biological samples than environmental samples were gathered.	It would be possible to compile more reliable and scientifically sound datasets to highlight regional CKDu similarities and differences with greater cooperation and harmonization of CKDu risk factor research methods. Understanding the origins of this fatal noncommunicable disease might be advanced by the detection of regionally distinct risk variables as opposed to worldwide risk factors.
Kadam et al., 2021, "Clinicopathological and Biochemical Profile of Chronic Kidney Disease of Unknown Aetiology in a Tertiary Care Rural Hospital of Central India" [30].	Diabetes or hypertension, young and middle-aged people who live in rural communities, and drinking well water are the main risk factors for the condition.	The study made clear the importance of reviewing health regulations and allocating funds for the prevention and treatment of CKDU in Maharashtra, Central India.

Therapy options in CKDu

Lifestyle medication, less use of chemicals/pesticides, and proper hydration should be encouraged among the population through awareness programs at the local level as well as with the help of the government. There should be awareness that prevention is better than cure. Renal replacement therapy, like renal transplantation, is undoubtedly the best option for diseased people. Similar-area eligible donors may be at risk of contracting CKDu and require strict observation after donating organs. The preferable choice is peritoneal dialysis (PD). An innovative program in Sri Lanka called "PD First" teaches neighborhood doctors to provide PD to clients with late CKDu. The Aarogyasri insurance program in Telangana and Andhra Pradesh enables underprivileged clients to receive free hemodialysis and transplantation in public and corporate institutions [32].

A well-rounded diet, adequate glycemic control, lipid management, moderate protein restriction, and salt restriction are all proven to stop the advancement of CKD. An excess of phosphorus in those with chronic kidney disease reduces calcium levels, compromising the bones and increasing the risk of fracture. Bone, as well as joint pain, along with itching, can be brought on by elevated phosphorus levels [33].

Due to the great burden of vascular, diabetic, and hypertension disorders, the incidence of non-cancerous ailments such as chronic kidney disease is steadily rising. The general public may only adopt a good outlook and a healthy way of life for the prevention of CKD when they are adequately informed about the condition. Populations that are educated and knowledgeable are less likely to develop or advance to CKD [34].

Individuals with CKD should be particularly concerned about anemia because it is a sign of the progression of the condition, especially iron deficiency. There are a few treatment options now available for anemia in these individuals using intravenous iron (IV) therapy. In terms of the criterion of iron deficiency anemia, IV iron sucrose therapy has been proven to be more beneficial, acceptable, and efficacious in individuals with CKD than oral iron treatment [35].

## Conclusions

Research should focus on developing specific biomarkers to detect CKDu in the very early stages of the condition so that intervention can be made to arrest the progression of end-stage renal disease (ESRD). The affected regions of the entire world should establish international research facilities that cooperate with numerous regional facilities by exchanging materials or conducting more extensive research. Vigilance, standardized disease records, and supervision systems are essential for reducing CKD and CKDu occurrences. By encouraging honest, transparent communication, the nation's ecological toxicology and epidemiology networks must be strengthened. Since CKDu mostly affects underdeveloped countries with limited resources, the WHO may declare it a global epidemic and increase funding for studies in these countries.
